# PD-L1 expression correlates with the oncological severity and prognosis of early-stage lung cancer

**DOI:** 10.1007/s00595-025-03070-6

**Published:** 2025-06-04

**Authors:** Ken Onodera, Hirotsugu Notsuda, Sakiko Kumata, Tatsuaki Watanabe, Yui Watanabe, Takaya Suzuki, Takashi Hirama, Hisashi Oishi, Yoshinori Okada

**Affiliations:** Department of Thoracic Surgery, Institute of Development, Aging and Cancer, Tohoku University, Seiryomachi 4-1, Aoba-ku, Sendai, Miyagi 980-8575 Japan

**Keywords:** Lung cancer, NSCLC, PD-L1, Expression, Malignancy

## Abstract

**Purpose:**

The expression of PD-L1 is linked to lung cancer severity; however, its prognostic value after resection is unclear. In this study, we investigated its role in resected lung cancers.

**Methods:**

We analyzed 658 patients with stage pIA–IIIA NSCLC who underwent complete resection. We assessed the PD-L1 expression by stage and its link to cancer severity, focusing further on its prognostic impact in resected stage I cell lung cancer.

**Results:**

The high expression of PD-L1 increased with disease progression (13.0% in IA to 36.2% in III). In stage I non-small cell lung cancer, elevated PD-L1 expression levels were more common in patients with serum CEA levels ≥ 5 (26.0%), SUVmax ≥ 5 (26.7%), and squamous cell carcinoma (41.5%). PD-L1-negative patients showed a better prognosis than PD-L1-positive patients, even with the use of immune checkpoint inhibitors following relapse (5-year OS: 94.3% vs. 83.2%, p < 0.01).

**Conclusion:**

The expression of PD-L1 in lung cancer appears to be associated with oncological severity and may influence the prognosis of early-stage disease. Additionally, in early-stage lung cancer, immune checkpoint inhibitors may not fully compensate for the negative prognostic impact of the high expression of PD-L1.

**Supplementary Information:**

The online version contains supplementary material available at 10.1007/s00595-025-03070-6.

## Introduction

Non-small cell lung cancer (NSCLC) is the leading cause of cancer-related death worldwide. The 5-year overall survival (OS) rates for patients with resected lung cancer according to stage are as follows: stage IA, 84%; IB, 73%; IIA, 65%; IIB, 56%, and IIIA, 41%, indicating a substantial potential for improvement [1]. As a significant proportion of lung cancer patients who undergo resection experience relapse even with stage I disease, identifying those at high risk of recurrence and establishing effective perioperative treatments is crucial.

The effectiveness of various immune checkpoint inhibitors (ICIs), including the programmed cell death protein 1 (PD-1) inhibitor nivolumab (Ono Pharmaceutical, Osaka, Japan), has recently been established, particularly in advanced lung cancer [2]. This development has extended to perioperative treatment for NSCLC, as evidenced by several trials, including Impower010 and Checkmate816, which have reported favorable outcomes [3–5]. Consequently, ICI therapy has become a standard approach for the perioperative treatment of resectable stage II-IIIA NSCLC.

The expression of programmed cell death ligand 1 (PD-L1) in cancer tissues serves as a biomarker for ICI efficacy [6]. As the expression of PD-L1 increases, so does the efficacy of ICIs. Furthermore, recent reports have suggested a correlation between the expression of PD-L1 and the oncological prognosis of lung cancer [7–9]. However, the impact of the expression of PD-L1 on the prognosis of early-stage resected lung cancer and the implications for perioperative ICI therapy remain unclear. This study aimed to assess the PD-L1 expression in early-stage lung cancer and its prognostic significance.

## Methods

### Ethical statement

The institutional review board at Tohoku University Hospital in Sendai, Japan approved this retrospective review of a prospective database (approval date: September 28, 2020; approval code: 2021-1-912-1). The requirement for informed consent was waived because of the retrospective nature of the study.

### Patients

**Fig. 1:**
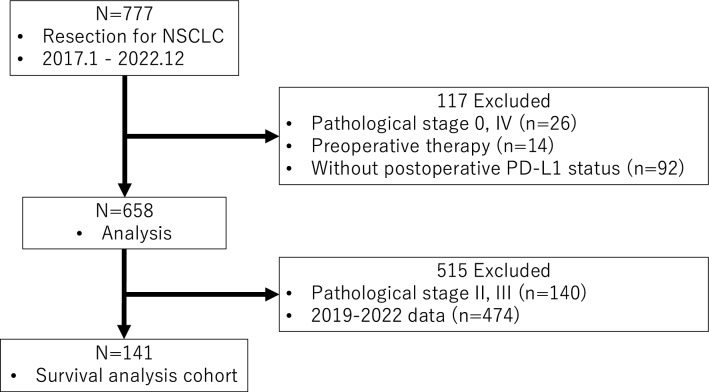
Cohort diagram

Between January 2017 and December 2022, 777 patients underwent lung resection for NSCLC at Tohoku University Hospital. Patients with pathological stages I-III were included in this study, excluding those who received preoperative therapy or had insufficient data on the postoperative PD-L1 status. Six hundred fifty-eight patients were excluded (Fig. 1). The choice of surgical procedure was guided by the treating physician, following the lung cancer treatment guidelines. Postoperatively, patients were routinely assessed at three-month intervals for the first two years and typically at six-month intervals thereafter. Follow-up evaluations included a physical examination, chest radiography, blood tests (including tumor markers), and chest computed tomography (CT). Additional assessments, such as brain magnetic resonance imaging (MRI) and positron emission tomography (PET)/CT scans, were performed based on any symptoms or signs of recurrence. The policy was to maintain follow-up for at least five years postoperatively. Recurrence was diagnosed through physical examination and imaging, and histological confirmation was obtained when clinically indicated.

### Clinicopathologic evaluations

All patient data were extracted from the electronic medical records and internal databases. Clinical and pathological staging were reassessed based on the eighth edition of the TNM classification [1]. Histological typing followed the World Health Organization classification [10]. The expression of PD-L1 was evaluated in formalin-fixed, paraffin-embedded tumor samples obtained by surgical resection using PD-L1 IHC 22C3 pharmDX assay. The cutoff values for the expression of PD-L1 were defined as follows: ≥ 50% (high expression), 1–49% (low expression), and 0% (negative).

### Statistical analysis

We examined PD-L1 expression across various pathological stages and specifically analyzed the features of patients with high PD-L1 expression levels in the stage I group. Additionally, we explored the relationship between PD-L1 status and survival outcomes in patients with stage I NSCLC using data from patients who underwent lung resection between 2017 and 2018. OS was defined as the time from surgery to death from any cause or last follow-up data. Recurrence-free survival (RFS) was defined as the time from surgery to recurrence, death from any cause, or censorship at the final follow-up. Ipsilateral intrathoracic and mediastinal recurrences were defined as local recurrence. Other recurrence was considered to be distant recurrence. Patient characteristics were summarized using frequencies and descriptive statistics including medians and ranges. OS and RFS estimates were calculated using the Kaplan–Meier method. Hazard ratios (HRs) and confidence intervals (CIs) were estimated using univariate and multivariate proportional hazards models.

All statistical analyses were conducted using JMP Pro (ver. 17.1.0, SAS Institute, Cary, NC, USA). Statistical significance was set at P < 0.05, without adjustment for multiple comparisons.

## Results

### Patient characteristics

Table 1 presents the clinicopathological characteristics of the patients. The cohort consisted of 389 (59.1%) males and 269 (40.9%) females, with median age of 71 (interquartile range 65 to 76 years) years. Among the patients, 414 (62.9%) were ever-smokers and 524 (79.6%) had adenocarcinoma. The distribution of the pathological stages was as follows: stage I, n = 518 (78.7%); stage II, n = 82 (12.5%); and stage III, n = 58 (8.8%). The median serum carcinoembryonic antigen (CEA) level was 2.9 (interquartile range: 1.9 to 4.5), while the median maximum standardized uptake value (SUVmax) was 4.6 (interquartile range: 2.1 to 9.8). Additionally, 152 (23.1%) patients exhibited pleural invasion, 243 (36.9%) exhibited vascular invasion, 145 (22.0%) exhibited lymphatic invasion, and 19 (2.9%) had pulmonary metastases. The PD-L1 expression was categorized as high in 128 (19.5%) patients, low in 268 (40.7%) patients, and negative in 262 (39.8%) patients.

**Table 1:** Patient characteristics in the entire cohort

Factors		All patients (n = 658)
Age (years)	Median (IQR)	71 (65–76)
Sex	Male	389 (59.1%)
	Female	269 (40.9%)
Smoking history	Never	244 (37.1%)
	Ever	414 (62.9%)
Serum CEA level	Median (IQR)	2.9 (1.9–4.5)
SUVmax	Median (IQR)	4.6 (2.1–9.8)
Histology	Adenocarcinoma	524 (79.6%)
	Squamous cell carcinoma	99 (15.0%)
	Others	35 (5.3%)
Total size (mm)	Median (IQR)	21 (14–28)
Invasive size (mm)	Median (IQR)	18 (12–27)
Pathological stage	IA	414 (62.9%)
	IB	104 (15.8%)
	II	82 (12.5%)
	III	58 (8.8%)
Pleural invasion	None	499 (75.8%)
	Present	152 (23.1%)
Vascular invasion	None	413 (62.8%)
	Present	243 (36.9%)
Lymphatic invasion	None	511 (77.7%)
	Present	145 (22.0%)
Pulmonary metastasis	None	638 (97.0%)
	Present	19 (2.9%)
PD-L1 expression	< 1%	262 (39.8%)
	1–49%	268 (40.7%)
	≥ 50%	128 (19.5%)

*IQR* interquartile range, *CEA* carcinoembryonic antigen, *SUVmax* maximal value of standardized uptake value, *PD-L1* programmed cell death ligand 1

Table 2 shows the clinicopathological characteristics of the stage I patients included in the survival analysis cohort. The group consisted of 77 (54.6%) males and 64 (45.4%) females, with a median age of 70 (interquartile range: 63.5 to 75) years. Eighty-one (57.4%) were smokers. Surgical intervention included lobectomy in 108 patients (76.6%). Histologically, 117 (83.0%) patients had adenocarcinoma. Pathological staging was classified as IA in 108 (76.6%) patients and IB in 33 (23.4%). Regarding the invasive characteristics, 20 (14.2%) patients showed pleural invasion, 38 (27.0%) showed vascular invasion, and 12 (8.5%) showed lymphatic invasion. The PD-L1 expression was categorized as high in 19 (13.5%) patients, low in 50 (35.5%) patients, and negative in 72 (51.1%) patients. Recurrence patterns included local recurrence in 8 (5.7%) patients, distant recurrence in 5 (3.5%), and both local and distant recurrence in 4 patients (2.8%). In patients who received limited resection, recurrence was observed in 2 patients with local recurrence in the PD-L1-positive and PD-L1-negative groups.

**Table 2:** Patient characteristics in survival analysis cohort

Factors		All patients (n = 141)		PD-L1 negative (n = 72)	PD-L1 positive (n = 69)	*p-value*
Age (years)	Median (IQR)	70 (63.5–75)		70 (62–75)	70 (65.5–75.5)	0.29
Sex	Male	77 (54.6%)		36 (50.0%)	41 (59.4%)	0.26
	Female	64 (45.4%)		36 (50.0%)	28 (40.6%)	
Smoking history	Never	60 (42.6%)		33 (45.8%)	27 (39.1%)	0.42
	Ever	81 (57.4%)		39 (54.2%)	42 (60.9%)	
Serum CEA level	Median (IQR)	2.8 (1.7–4.6)		2.4 (1.1–3.7)	3 (2–5.2)	0.82
SUVmax	Median (IQR)	2.6 (1.5–5.5)		2 (1.4–3.6)	3.8 (1.5–7.0)	< 0.01
Operation	Lobectomy	108 (76.6%)		53 (73.6%)	56 (81.2%)	0.49
	Pneumonectomy	1 (0.7%)		1 (1.4%)	0	
	Limited resection	32 (22.7%)		18 (25.0%)	15 (21.7%)	
Histology	Adenocarcinoma	117 (83.0%)		71 (98.6%)	46 (66.7%)	< 0.01
	Squamous cell carcinoma	18 (12.8%)		1 (1.4%)	17 (24.6%)	
	Others	6 (4.3%)		0	6 (8.7%)	
Total size (mm)	Median (IQR)	19 (12.5–26)		19 (12–25)	17 (13–28)	0.95
Invasive size (mm)	Median (IQR)	13 (9–22)		12 (6–21)	15 (9–25)	0.05
Pathological stage	IA	108 (76.6%)		62 (86.1%)	46 (66.7%)	< 0.01
	IB	33 (23.4%)		10 (13.9%)	23 (33.3%)	
Pleural invasion	None	121 (85.8%)		66 (91.7%)	55 (79.7%)	0.04
	Present	20 (14.2%)		6 (8.3%)	14 (20.3%)	
Vascular invasion	None	103 (73.0%)		61 (84.7%)	42 (60.9%)	< 0.01
	Present	38 (27.0%)		11 (15.3%)	27 (39.1%)	
Lymphatic invasion	None	129 (91.5%)		70 (97.2%)	59 (85.5%)	< 0.01
	Present	12 (8.5%)		2 (2.8%)	10 (14.5%)	
PD-L1 expression	< 1%	72 (51.1%)		72 (100%)	0	N/A
	1–49%	50 (35.5%)		0	50 (72.5%)	
	≥ 50%	19 (13.5%)		0	19 (27.5%)	
Recurrence	None	124 (87.9%)		66 (91.7%)	58 (84.1%)	N/A
	Local	8 (5.7%)		3 (4.2%)	5 (7.2%)	
	Distant	5 (3.5%)		0	5 (7.2%)	
	Both	4 (2.8%)		3 (4.2%)	1 (1.4%)	

*IQR* interquartile range, *CEA* carcinoembryonic antigen, *SUVmax* maximal value of standardized uptake value, *PD-L1* programmed cell death ligand 1

### PD-L1 expression

**Fig. 2:**
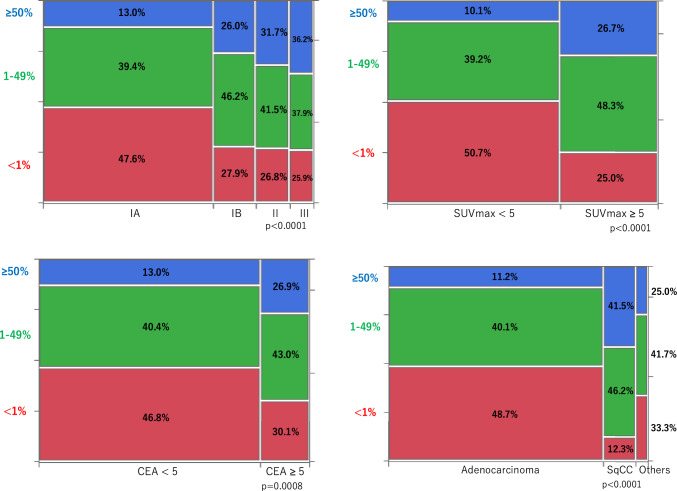
Relationship between clinicopathological factors and PD-L1 expression. Stage (**A**). SUVmax (**B**). Serum CEA level (**C**). Histology (**D**). The vertical and horizontal axes of the graph show the percentages of patients

Across the entire cohort, 41.7% patients were PD-L1-negative, 40.7% exhibited low expression levels, and 19.5% showed high expression levels. When analyzed by pathological stage, the percentage of PD-L1-negative tumors decreased with the progression of the disease stage, as follows: stage IA, 47.6%; IB, 27.9%; II, 26.8%; and III, 25.9%. Conversely, the percentage of tumors with high PD-L1 expression levels increased as the disease progressed, as follows: stage IA, 13.0%; IB, 26.0%; II, 31.7%; and III, 36.2% (Fig. 2A).

In the subgroup of patients with pathological stage I, high PD-L1 expression levels were more prevalent in patients with SUVmax ≥ 5 (SUVmax ≥ 5 vs. < 5: 26.7% vs. 10.1%) (Fig. 2B) and in those with serum CEA levels ≥ 5 (CEA ≥ 5 vs CEA < 5: 26.0% vs. 13.0%) (Fig. 2C). Additionally, high PD-L1 expression levels were detected in 11.2% of adenocarcinomas and 41.5% of squamous cell carcinomas (Fig. 2D).

### Survival analysis

**Fig. 3:**
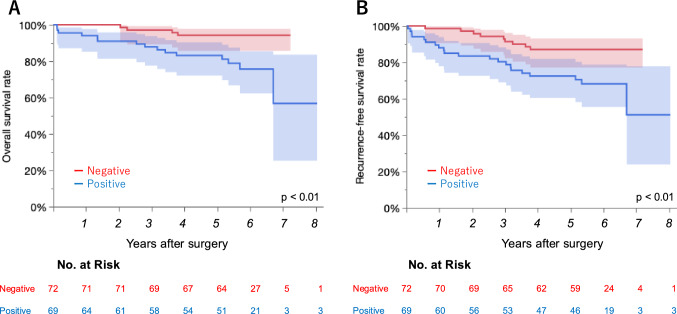
Overall survival (**A**) and recurrence-free survival (**B**) according to the expression of PD-L1 in stage I lung cancer

**Fig. 4:**
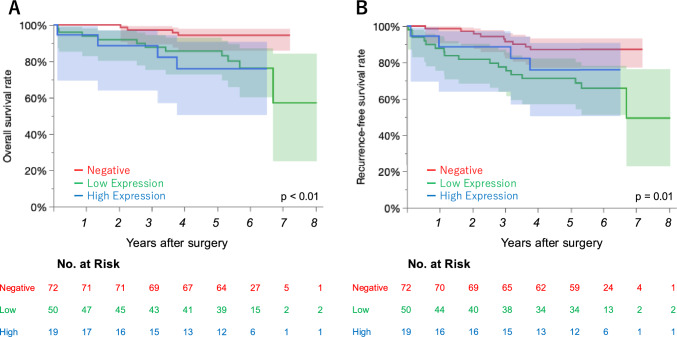
Overall survival (**A**) and recurrence-free survival (**B**) according to the expression of PD-L1 in stage I lung cancer

We conducted a survival analysis of 141 patients with pathological stage I NSCLC who underwent adequate follow-up. The median follow-up period was 5.6 years for all patients and 5.7 years for censored patients. Patients with PD-L1-negative disease showed better OS and RFS than those with PD-L1-positive disease (5-year-OS: 94.3% vs. 83.2%, p < 0.01; 5-year RFS: 87.1% vs. 72.5%, p < 0.01) (Fig. 3). No significant differences in OS and RFS were observed between patients with low and high PD-L1 expression levels (low vs. high: 5-year OS: 85.6% vs. 75.9%, p = 0.68; 5-year RFS: 71.2% vs. 75.9%, p = 0.52) (Fig. 4). Among the 5 cases with PD-L1-positive recurrent disease without driver mutations, 2 patients could not be treated after recurrence (Table 3). For reference, the survival curves according to the PD-L1 expression status for patients at all stages and stages II–III are also shown in Supplemental Fig. 1.

**Table 3:** Treatment after recurrence

Case	Age at surgery	Sex	pStage	PD-L1 status	Driver mutation	Recurrence type	Treatment after recurrence
1	81	Female	IB	Negative	Negative	Both	BSC
2	75	Male	IA3	Negative	Negative	Local	Radiotherapy
3	71	Female	IB	Low expression	EGFR del19	Local	TKI
4	75	Male	IA1	Negative	Negative	Local	Chemotherapy
5	68	Female	IB	Low expression	Negative	Distant	ICI
6	82	Male	IA3	Low expression	Negative	Local	Chemotherapy
7	62	Female	IB	Low expression	EGFR del19	Distant	TKI
8	65	Male	IB	Negative	Negative	Local	ICI
9	65	Male	IB	Low expression	EGFR L858R	Distant	TKI, ICI
10	47	Female	IA2	Low expression	Negative	Local	TKI
11	66	Female	IA1	Low expression	EGFR del19	Local	TKI
12	69	Male	IA3	Low expression	EGFR del19	Both	TKI
13	67	Male	IB	Low expression	EGFR del19	Local	TKI
14	76	Male	IB	Low expression	Negative	Distant	BSC
15	80	Male	IB	High expression	Negative	Distant	BSC
16	76	Male	IA2	Negative	Negative	Both	ICI
17	70	Female	IB	Negative	EGFR Ex20Ins	Both	Chemotherapy

*PD-L1* programmed cell death ligand 1, *EGFR* epdermal growth factor receptor, *BSC* best supportive care, *TKI* tyrosine kinase inhibitor, *ICI* immune checkpoint inhibitor

In the multivariate analysis of OS, the expression of PD-L1 was not identified as an independent prognostic factor; smoking history was the only factor that showed statistical significance (Table 4).

**Table 4:** Multivariate analysis for overall survival

Factors		HR (95% CI)	*p-*value
Age (years old)		1.103 (1.019–1.208)	N/A
Sex	Male		
	Female	2.470 (0.369–16.547)	0.35
Smoking history	Never		
	Ever	12.918 (1.124–148.483)	0.04
Serum CEA level	< 5 ng/mL		
	≥ 5 ng/mL	1.239 (0.358–4.283)	0.74
SUVmax	< 5		
	≥ 5	2.281 (0.542–9.598)	0.26
Histology	Adenocarcinoma		
	Others	1.066 (0.272–4.183)	0.93
Pathological stage	Stage IA		
	Stage IB	1.497 (0.485–4.617)	0.48
Vascular invasion	None		
	Present	1.012 (0.302–3.387)	0.98
Lymphatic invasion	None		
	Present	2.395 (0.663–8.653)	0.18
PD-L1 expression	Negative		
	Positive	4.129 (0.758–22.483)	0.10

*IQR* interquartile range, *CEA* carcinoembryonic antigen, *SUVmax* maximal value of standardized uptake value, *PD-L1* programmed cell death ligand 1, *N/A* not available

## Discussion

In our study, the PD-L1 expression levels tended to increase with the progression of pathological stage in patients with lung cancer who underwent surgical resection. This finding aligns with Sun et al.’s 2016 report that linked the expression of PD-L1 to pathological stage [7], supporting the notion that the expression of PD-L1 may increase as the disease progresses. Several subsequent studies, albeit limited in number, have suggested a correlation between the expression of PD-L1 and disease stage [11], and our analysis reinforces these observations in a substantial case series, specifically 658 cases of resected lung cancer. Biological studies have shown that PD-1 and PD-L1 regulate T-cell activity [12], and it is thought that cancers exploit PD-L1/PD-1 signaling to escape T-cell immunity. This may explain the clinical association between the expression of PD-L1 and the severity of cancer.

In our study, we further discovered that the expression of PD-L1 was associated with serum CEA levels, the SUVmax of the primary tumor, and histology (adenocarcinoma vs. squamous cell carcinoma) in stage I patients. Motono et al. also reported an association between PD-L1 and both serum CEA levels and SUVmax [11]; however, their study was not confined to early-stage lung cancer, suggesting that stage may influence the expression of PD-L1. Contradictory findings exist regarding the relationship between the expression of PD-L1 and the histological type. D'Incecco et al. reported that the rate of PD-L1 positivity was higher in adenocarcinoma than in squamous cell carcinoma [13]. On the other hand, Schmidt et al. reported that the expression of PD-L1 was higher in squamous cell carcinoma than in non-squamous cell carcinoma [14]. Our analysis, focused solely on early-stage lung cancer, showed that in addition to stage effects, the expression of PD-L1 correlates with serum CEA levels, the SUVmax of the primary tumor, and histology. These factors are known to be linked to the prognosis of lung cancer [15–18], indicating that the expression of PD-L1 is associated not only with disease stage but also with the grade of malignancy.

Importantly, our findings suggest that patients with stage I NSCLC who are PD-L1-negative have a better prognosis after resection than those who are PD-L1-positive. Although there was a mix of lobectomies and limited resections in this study, the number of cases of recurrence among the 32 cases of limited resection was not biased and did not appear to influence group differences. Previous studies have explored the relationship between the expression of PD-L1 and the prognosis [7, 19], but the results are controversial. Given that the expression of PD-L1 tends to increase as the grade of malignancy advances, it is theoretically plausible that PD-L1-negative patients would experience better outcomes, as observed in our study. Of note in this study was the favorable prognosis of PD-L1-negative patients, even when limited to stage I patients. In our study, the use of ICIs after relapse has become a standard practice, potentially influencing prognostic outcomes. Patients with PD-L1-negative lung cancer had a better prognosis than PD-L1-positive patients, despite the lack of benefit of ICI treatment after recurrence. Among the cases involving recurrent PD-L1-positive disease without driver gene mutations in this study, two of five patients could not be treated after recurrence due to disease progression. The two patients had a poor performance status due to brain metastasis and meningeal dissemination, respectively, and ICIs could not be introduced. It is possible that cases of PD-L1-positive lung cancer did not reach the treatment stage due to rapid progression. This observation indicates that the potential disadvantages associated with the expression of cannot be mitigated by ICI therapy. Therefore, a subset of stage I NSCLC that is PD-L1-positive may be a target for perioperative treatment strategies using ICI. Unlike the results of the univariate analysis, in the multivariate analysis, PD-L1 positivity was not an independent prognostic factor (HR 4.129, 95% CI 0.758–22.483, p = 0.10), although there was a trend towards higher HR. This may be due to the insufficient number of analyzed cases.

### Limitations

This study had several limitations. First, it was retrospective in nature; therefore, a selection bias could not be entirely eliminated. Moreover, the prognostic cohort included only 141 cases, yielding less statistical power than the analysis of the entire cohort. The lack of significant differences in the multivariate analysis, despite significant differences in the univariate analysis, may be due to low statistical power. Owing to these limitations, a prospective observational study may be necessary for a more accurate evaluation. Second, this study was conducted at a single center. To confirm the relationship between the PD-L1 expression and lung cancer malignancy grade, validating the results across multiple centers is essential.

## Conclusions

This study suggests a relationship between the expression of PD-L1 and lung cancer severity. Additionally, patients with stage I lung cancer who are PD-L1-positive may have a worse prognosis than those who are PD-L1-negative, and ICI therapy may not compensate for this disadvantage. These patients may be targets for the development of optimal perioperative treatment strategies.

## Supplementary Information

Below is the link to the electronic supplementary material.

Supplementary file1 (PDF 241 KB)

## Abbreviations

NSCLC: Non-small cell lung carcinoma
OS: Overall survival
ICI: Immune checkpoint inhibitor
PD-1: Programmed cell death protein 1
PD-L1: Programmed cell death ligand 1
CT: Computed tomography
MRI: Magnetic resonance imaging
PET: Positron emission tomography
RFS: Recurrence-free survival
HR: Hazard ratio
CI: Confidence interval
CEA: Carcinoembryonic antigen
SUVmax: Maximal standardized uptake value
