# Supplementary material for: PD-L1 expression correlates with the oncological severity and prognosis of early-stage lung cancer
Source: Surg Today. 2025 Jun 4;55(11):1635–43. doi: 10.1007/s00595-025-03070-6 (PMC12534232; doi:10.1007/s00595-025-03070-6)
Supplement: Supplementary file 1 — Supplementary file1 (PDF 241 KB) [file 595_2025_3070_MOESM1_ESM.pdf]

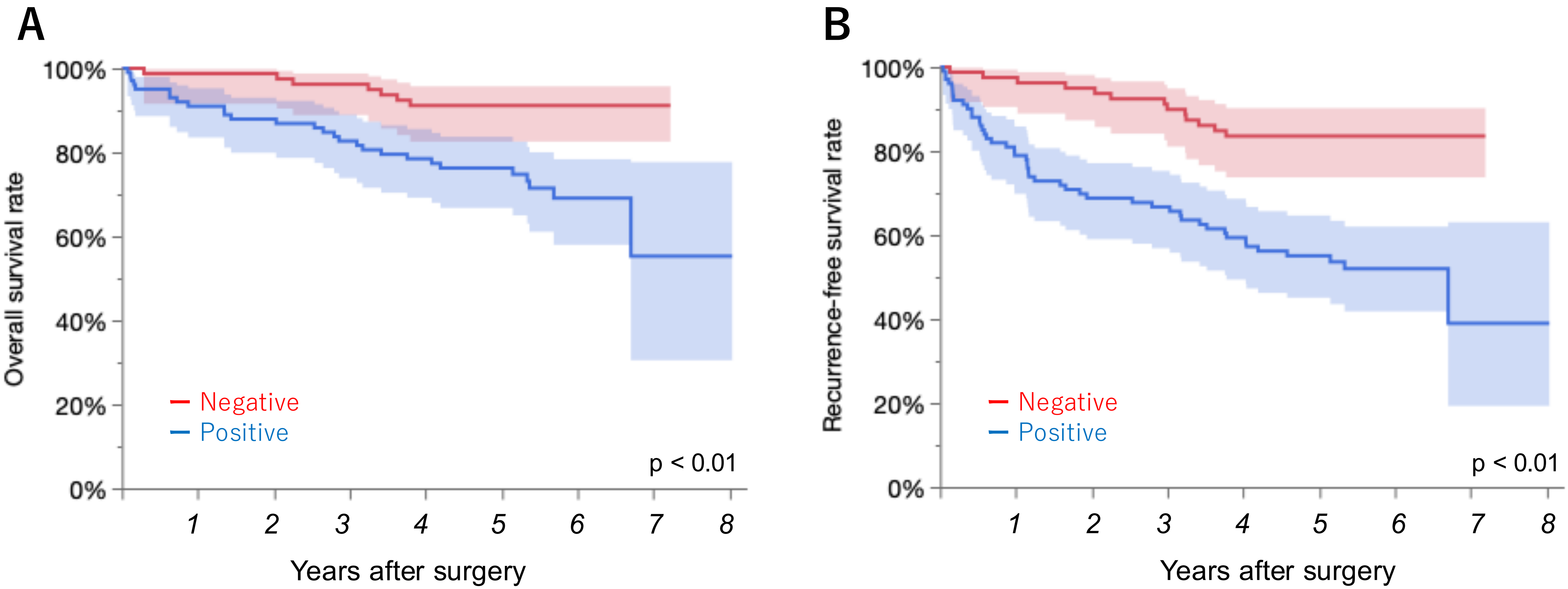

No. at Risk

Negative	81	79	79	77	73	70	30	5	1
Positive	102	90	86	80	73	63	23	2	2

No. at Risk

Negative	81	78	76	73	67	64	27	4	1
Positive	102	79	68	65	57	50	19	2	2

Supplement Figure 1

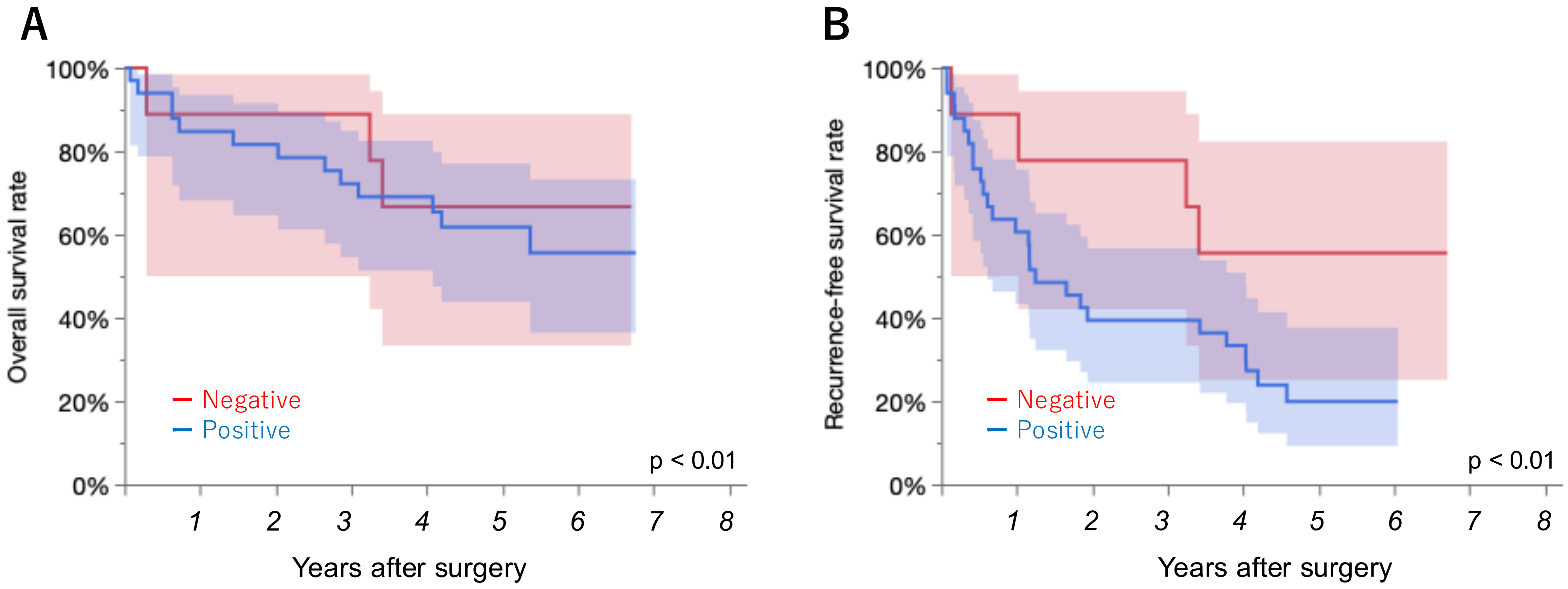

No. at Risk (A)

Negative	9	9	9	9	7	7	4	1	1
Positive	33	28	27	24	21	14	4	1	1

No. at Risk (B)

Negative	9	9	8	8	6	6	4	1	1
Positive	33	21	14	14	12	6	2	1	1

Supplement Figure 2
